# YOLO11m-cls applied to sex and age classification based on the radiographic analysis of the nasal aperture

**DOI:** 10.1038/s41598-025-24593-5

**Published:** 2025-11-19

**Authors:** Leonardo Scavassini, Rianne Silva, Amber Khan, Wahaj Anees, Jared Murray, Nikolaos Angelakopoulos, Marianna Arakelyan, Lucas Porto, André Abade, Ademir Franco

**Affiliations:** 1 Division of Forensic Dentistry, Faculdade São Leopoldo Mandic, Campinas, São Paulo, Brazil; 2 Private practice, consultant, Dundee, Dundee, UK; 3 Department of Orthodontics and Dentofacial Orthopedics, University of Bern, Freiburgstrasse 7, Bern, 3010 Switzerland; 4 Department of Therapeutic Stomatology, Institute of Dentistry, Sechenov University, Moscow, Russia; 5 Private practice, consultant, Brasília, Brazil; 6 Computer Science, Federal Institute of Science and Technology, Barra do Garças, Brazil

**Keywords:** Anatomy, Artificial intelligence, Forensic dentistry, Nasal aperture, Radiology, Anatomy, Computational biology and bioinformatics, Health care, Mathematics and computing, Medical research

## Abstract

Deep learning tools based on computer vision have emerged as alternative methods for assessing radiographic image patterns. These approaches have been explored for various forensic applications, including sex and age estimation. This study aimed to evaluate the diagnostic accuracy of a Convolutional Neural Network (CNN) in classifying radiographic images by sex and age, focusing on the nasal aperture as the morphological feature of interest. The sample comprised 9,349 radiographs annotated for the nasal aperture region. A CNN architecture based on the You Only Look Once series—specifically the intermediate version 11 for object classification (YOLO11m-cls)—was implemented, with training performed using 5-fold cross-validation. The overall accuracy rate was 74% (ranging from 61% to 88%), and the area under the Receiver Operating Characteristic (ROC) curve was 0.74. Correct classification rates were 73% for males and 75.17% for females. Accuracy varied with age, showing a 10% decrease among younger individuals compared to older ones. The study confirmed the reduced expression of sexually dimorphic traits in younger individuals and supported existing recommendations against performing sex estimation in subadults. Within the present methodological framework, the nasal aperture demonstrated limited applicability for sex estimation in the studied sample, with an accuracy rate corresponding to approximately one misclassification out of every four predictions.

**Supplementary Information:**

The online version contains supplementary material available at 10.1038/s41598-025-24593-5.

## Introduction

In forensic science, human identification often relies on skeletal and dental morphological features to reconstruct a postmortem biological profile^1^. This profile can be composed of the estimated sex, age, population affinity, and stature of an individual^2^. Modern forensic anthropology has benefited from computer vision and artificial intelligence technologies to enhance and optimize its practices^3^. Some of these resources rely on the virtual analysis of the human anatomy by means of three-dimensional (3D) and bidimensional (2D) medical imaging, such as computed tomography^4,5^ and traditional radiography^6,7^ respectively. While the former allows realistic and detailed visualization of bones and teeth,^8^ the latter offers the advantages of faster data processing and transfer, as well as the ability to acquire and store large samples in small spaces. These advantages are especially useful when operating computer vision solutions via Convolutional Neural Network (CNN). This is because CNNs are deep learning models designed to analyze visual data through successive layers that detect image patterns,^9^ enabling complex feature classification and detection. Recent forensic science studies employing CNN to detect and classify anatomical features in radiographs have demonstrated applicability of artificial intelligence for sex and age estimation^10–15^. Among the improvements foreseen for forensic practice, reduction in operator interventions and faster processing are expected^7^.

The nasal aperture is a maxillofacial region of interest when it comes to reconstructing a biological profile given its potential populational-^16^ and sex-specific^19,20^ variations. For instance, authors^19^ have demonstrated statistically significant differences between males and females based on the morphometric analyses of the nasal width and height in computed tomography scans. However, the mean accuracy rates were below 65%.^19^ Sexual dimorphism has also been reported in the scientific literature when measurements are taken directly from dry human skulls^20^. In this context, nasal height has been highlighted as a sexually dimorphic anatomical feature, being greater in males than in females^20^. Regarding shape, authors have found an absence of sexual dimorphism in the nasal aperture in specific populations, namely Black and White South African skulls^21^. Anthropological studies such as these have contributed to the current body of knowledge in the field. However, subsequent investigations can be proposed to further expand the scientific understanding of the nasal aperture’s applicability for sex estimation. To this end, radiographs of the viscerocranium may serve as valuable resources to facilitate large-scale data collection and analytical compatibility with CNN models.

Based on the foregoing, this study aimed to test the diagnostic accuracy of the nasal aperture for sex estimation using a semi-automated CNN approach applied to radiographic analysis.

## Materials and methods

### Study design and ethical aspects

In this study, a diagnostic accuracy test was designed to compare the performance of a CNN (index test) in estimating the sex of children, adolescents and young adults through analysis of the nasal aperture on panoramic radiographs. The assessment of medical images was conducted retrospectively using an existing database of patient records. All radiographs were acquired exclusively for diagnostic, therapeutic or dental treatment follow-up purposes, ensuring that no patient was exposed to ionizing radiation solely for research. The outlined investigation protocol received approval from the Institutional Committee of Ethics in Human Research at the Faculdade São Leopoldo Mandic (Protocol No. 76809023.9.0000.5374) and was reported in accordance with the Standards for Reporting of Diagnostic Accuracy Studies (STARD)^22^, while addressing current key considerations regarding dental artificial intelligence research^23^. The images utilized in this study constituted secondary data sourced from an established radiology database (Center of Oral Radiology and Imaging). Access to the data was authorized through informed permission granted by the database’s legal custodian. Given the retrospective design and the use of anonymized imaging data, the Institutional Committee of Ethics in Human Research at the Faculdade São Leopoldo Mandic formally waived the requirement for direct informed consent from individual patients.

## Participants

The sample consisted of panoramic radiographs (*n* = 9349) from Brazilian males (*n* = 4375, 46.8%) and females (*n* = 4974, 53.2%), aged between 6 and 22.9 years (Table 1). The inclusion criteria comprised individuals with at least one radiograph with known date of image acquisition, date of birth and recorded sex. The exclusion criteria included radiographs showing nose piercings, evidence of trauma or surgery in the middle third of the face (e.g., orthopedic fixation devices) or other metallic apparatus in the viscerocranium, or visible signs of skeletal deformity. Further dataset partitioning was performed at the participant level, meaning that only one image from each patient was assigned to a single split and not to another (i.e. train or validation), thereby preventing data leakage and promoting unbiased evaluation of model generalization.

**Table 1:** – Sample distribution by sex and age.

Age interval	Males	Females
6-6.99	284	297
7-7.99	341	338
8-8.99	292	333
9-9.99	301	330
10-10.99	200	198
11-11.99	210	184
12-12.99	200	212
13-13.99	195	203
14-14.99	199	208
15-15.99	198	223
16-16.99	271	200
17-17.99	200	200
18-18.99	200	196
19-19.99	216	212
20-20.99	417	584
21-21.99	307	517
22-22.99	344	539
**Total**	**4375**	**4974**

Age intervals expressed in years. Sample size (n): 9349, being 46.8% males and 53.2% females.

## Analysis

**Fig. 1:**
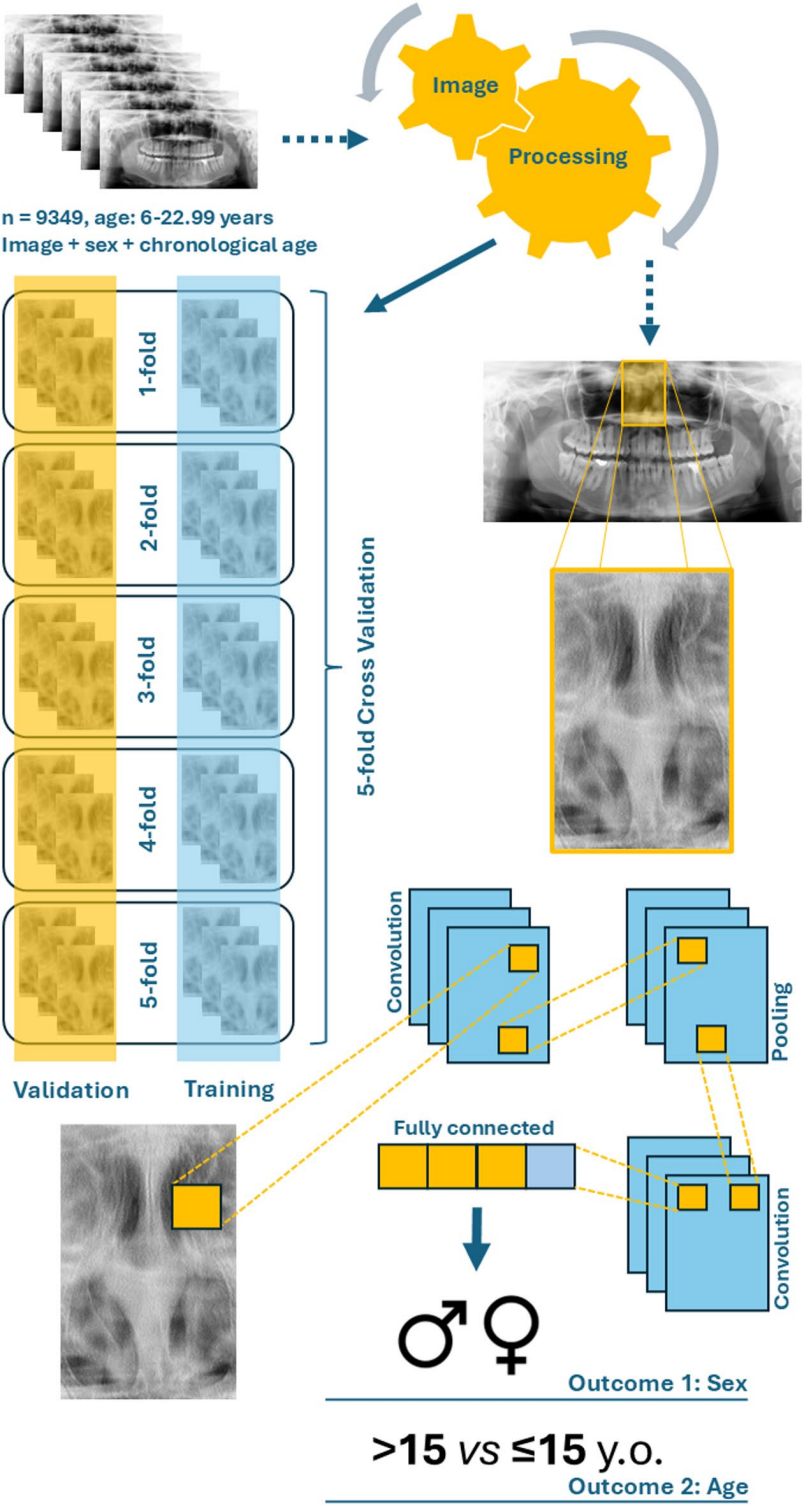
Workflow of a convolutional neural network (CNN) applied to panoramic radiographs for the estimation of sex and age. A total of 9349 radiographs from individuals aged from 6 to 22.99 years old (y. o.) were used. Images, sex, and chronological age were provided as input features. Data were split into five folds for cross-validation, with training (blue) and validation (yellow) sets. Image preprocessing was applied prior to CNN feature extraction through convolution and pooling layers. Fully connected layers integrated extracted features to predict two outcomes: sex (male [♂] vs. female [♀]) and age (> 15 years old vs. ≤ 15 years old).

Image anonymization was performed by cropping out the radiographic frames containing the patients’ age and sex and image side indicator (left/right). Subsequently, each radiograph was assigned an alphanumeric code to facilitate further de-identification. All radiographs were originally similar, as they were obtained from a single oral radiology clinic. However, to ensure higher standardization, they were pre-processed to preserve their size, image detail, spatial resolution, and quality. Image annotation was performed by five trained forensic odontologists experienced in annotations on panoramic radiographs^6,7,10,14^ using Darwin V7 software package (Darwin V7 Labs, London, UK) with its native bounding-box tool. The bounding-box enabled selection of the region of interest (ROI) on panoramic radiographs by manually dragging a rectangular outline over the image. In the present study, the ROI was the nasal aperture (Fig. 1), enabling a margin of 8–10% to preserve the nasal aperture contour and best fit its anatomic context. The images were resized to 224 × 224 pixels, converted to 3 channels (replication of the grayscale), scaled to the [0,1] range, and min–max normalized per image. Image augmentation was applied to the training dataset and included the following transformations with the indicated probabilities: random horizontal flip (*p* = 0.5), rotation of ± 7° (*p* = 0.5), translation of ± 6% (*p* = 0.3), zoom ranging from 0.9 to 1.1 (*p* = 0.3), brightness/contrast variation of ± 10% (*p* = 0.3), Gaussian noise with σ = 0.01 (*p* = 0.2) and mild sharpening (*p* = 0.2). Aggressive cropping was avoided to prevent truncation of the superior border of the ROI (nasal aperture region, often located close to the upper limit of the panoramic radiograph).

A deep learning architecture based on the YOLO11m-cls model^24,25^ was trained over 100 epochs (Table 2). Recent studies in the scientific literature have demonstrated the potential of this model series for medical imaging applications and have specifically supported its use in two-dimensional radiographic assessments^26^. This version of the model incorporates advanced architectural components, including the introduction of the C3k2 (Cross Stage Partial with kernel size 2) block, Spatial Pyramid Pooling – Fast (SPPF), and C2PSA (Convolutional block with Parallel Spatial Attention), which contribute to improving the model’s performance in several ways, such as enhanced feature extraction.

**Table 2:** Model architecture.

Component	Specifics
Backbone	YOLO11m^24,25^
Learning rate	0.01^25^
Optimizer	SGD (momentum: 0.937)^24^
Batch size	16
Epochs	100

Superscript numerals indicate references.

Categorical cross-entropy loss and L2 regularization (weight decay = 5 × 10⁻⁴) were used in the training, implemented in the stochastic gradient descent optimizer with a learning rate of 0.0125, momentum of 0.937, 100 epochs, and a batch size of 16. Model evaluation was performed with 5-fold cross-validation,^27,28^ where in each iteration approximately 20% of the images (*n* ≈ 1,869–1,870) were retained as an external test set, while from the remaining ~ 80% (*n* ≈ 7,479–7,480), about 10% was reserved exclusively for monitoring the training process. Early stopping was not applied; instead, at the end of 100 epochs, the checkpoint with the lowest monitoring loss was selected and subsequently evaluated on the corresponding test fold. We reported the average performance across all five folds. The choice of k = 5 represented a balance between computational cost and robustness: increasing k linearly raises the training cost (e.g., k = 10 would double the computational burden without proportionally improving precision), while with *n* = 9,349, each fold provided a sufficiently large test set to yield stable estimates and a training set large enough to preserve generalization. This arrangement ensured that all images were used once as test data, allowed confidence interval estimation from the distribution of fold scores, and provided a practically robust yet computationally feasible evaluation strategy. The computer vision analysis was performed by two experienced engineers.

## Test methods

The reference standards to which the CNN was compared were the individuals’ documented sex (binary: male or female) and their chronological age (obtained between the date of birth and date of image acquisition). YOLO11m-cls was tested based on its diagnostic performance to classify individuals according to sex after analyzing the nasal aperture in all the radiographs (combined sample). Binary classifications considered the decision cutoff of 0.5. Next, separate analyses were conducted to assess the diagnostic accuracy of the CNN by sex (correct classification of males and females) and by age group. In a subsequent experimental procedure, the CNN’s performance was exclusively evaluated for classifying individuals based on age (> 15 or ≤ 15 years). Multiclass task decisions considered the highest predicted probability. In this phase, the sample distribution was balanced with 4,403 individuals over 15 years and 4,946 individuals age 15 years younger. A subsequent age-based analysis was performed separately for males and females. Sample distribution was as follows: males over 15 years (*n* = 1,955), females over 15 years (*n* = 2,448), males aged 15 years or younger (*n* = 2,420), and females aged 15 years or younger (*n* = 2,526). It should be noted that this approach used the 15 years as the cut-off to distinguish younger and older individuals because the bony framework of the nose is estimated to grow more noticeably until around 15 years of age in males, and at an earlier age in females^29^. Moreover, this study acknowledges the significant limitations of sex estimation in subadult individuals and emphasizes that it should not be recommended in practice, particularly when using the nasal aperture as the evaluated parameter. Instead, the analysis of morphological features of viscerocranium and their differences between sexes across age groups was proposed and presented as an educational and exploratory approach, aiming to enhance basic anatomical understanding and supporting further research in the field.

To quantify the diagnostic accuracy of the nasal aperture in classifying individuals by sex and age, this study used metrics commonly employed to assess deep learning models in forensic computer-vision: accuracy, precision, recall, sensitivity and specificity. The outcomes were tabulated and visually presented using confusion matrices, Receiver Operating Characteristic (ROC) curves with their Area Under the Curve (AUC), and Gradient-weighted Class Activation Mapping (Grad-CAM). To account for variability across different datasets, mitigate overfitting, and ensure that performance metrics were not biased towards any specific part of the dataset, this study calculated the average of each metric across all five folds to obtain an overall measure of model performance. Computations were performed on a Linux machine running Ubuntu 22.04, equipped with an AMD Ryzen 9 7950X processor, 2 Nvidia^™^ RTX A5500 24 GB GPUs, and 128 GB of DDR5 RAM. All models were developed using TensorFlow API^30^ version 2.18. Python 3.8.10 was employed for algorithm implementation and data wrangling^31^.

## Results

When sex was considered a binary outcome for the CNN’s performance in analyzing the nasal aperture on panoramic radiographs, accuracy, precision, recall, sensitivity, and specificity all reached approximately 74%. For the combined sample, accuracy rates ranged from 61% to 88%.

**Fig. 2:**
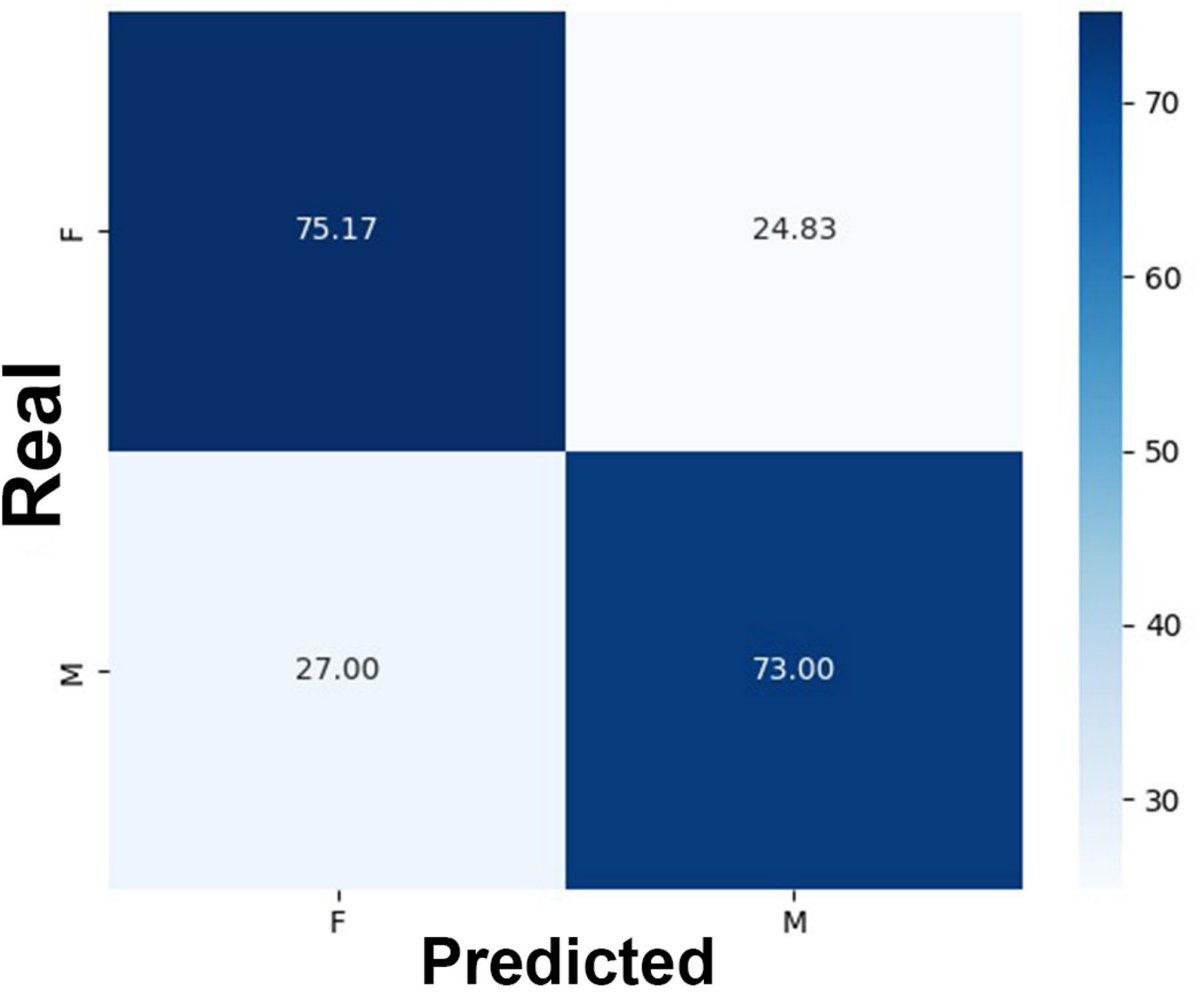
Confusion matrix comparing real (documental) and predicted sex of the present sample’s males (M) and females (F). Legend: The correct classification rate of males was 73%, while for females it was 75.17%.

**Fig. 3:**
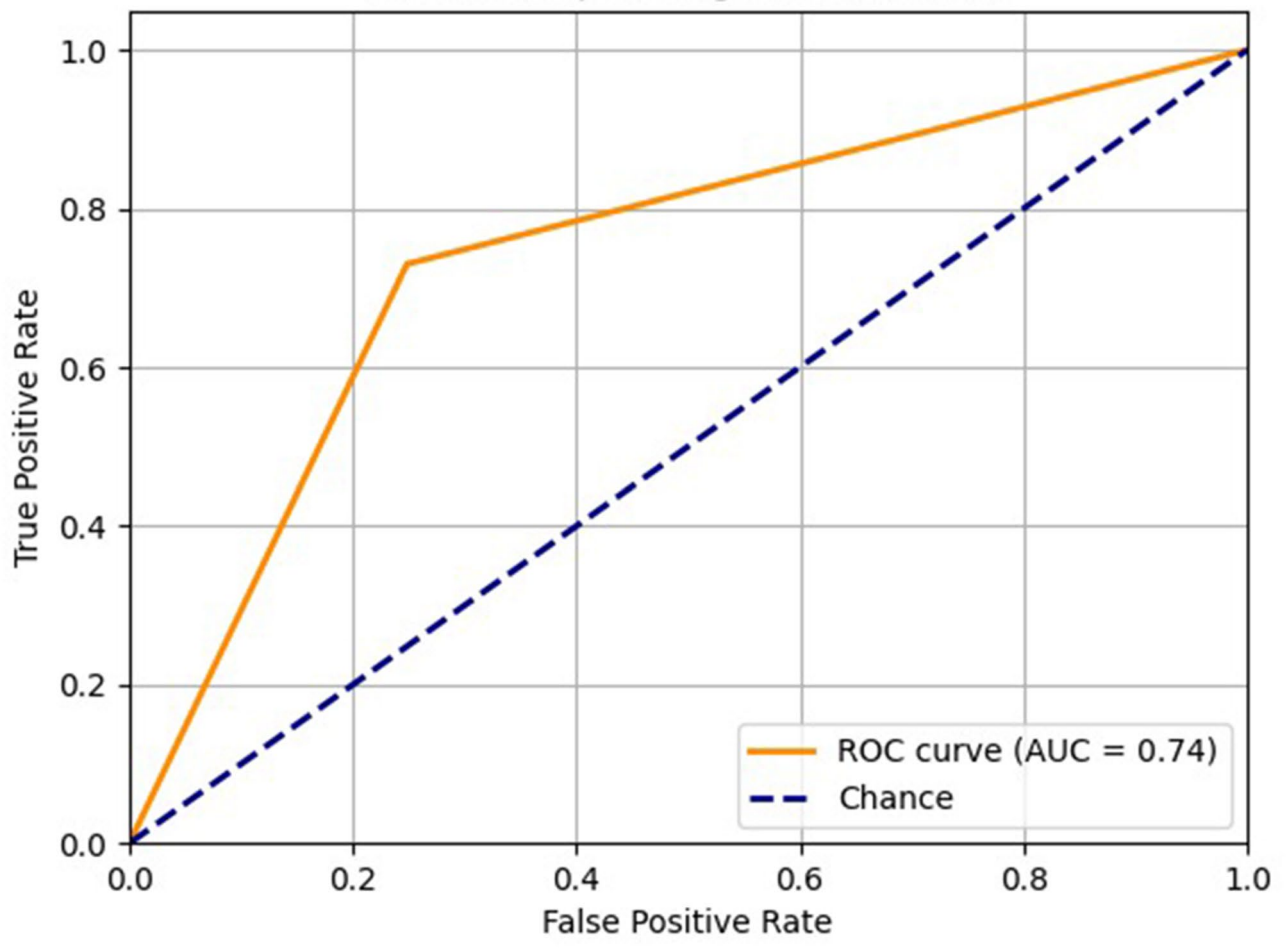
Receiver Operating Characteristic (ROC) curve for the assessment of sex. The Area Under the Curve (AUC) was 0.74 considering the true positive and false positive values.

In males, accuracy rates ranged from 50% to 90%, while in females they ranged from 54% to 86% (Table 3). The correct classification rate was 73% for males and 75.17% for females (Fig. 2). The area under the ROC curve was 0.74 (Fig. 3).

**Table 3:** Accuracy rates presented per age category and sex.

	Accuracy		
Age	Total	Males	Females
6-6.99	0.613333	0.684211	0.540541
7-7.99	0.680851	0.608696	0.750000
8-8.99	0.621951	0.627907	0.615385
9-9.99	0.724138	0.666667	0.777778
10-10.99	0.792453	0.807692	0.777778
11-11.99	0.645833	0.500000	0.818182
12-12.99	0.745763	0.709677	0.785714
13-13.99	0.800000	0.937500	0.684211
14-14.99	0.773585	0.750000	0.823529
15-15.99	0.796610	0.794118	0.800000
16-16.99	0.805556	0.837209	0.758621
17-17.99	0.886364	0.909091	0.863636
18-18.99	0.734694	0.781250	0.647059
19-19.99	0.650000	0.633333	0.666667
20-20.99	0.800000	0.767857	0.826087
21-21.99	0.800000	0.853659	0.770270
22-22.99	0.755556	0.684211	0.807692

Age categories expressed in years.

**Fig. 4:**
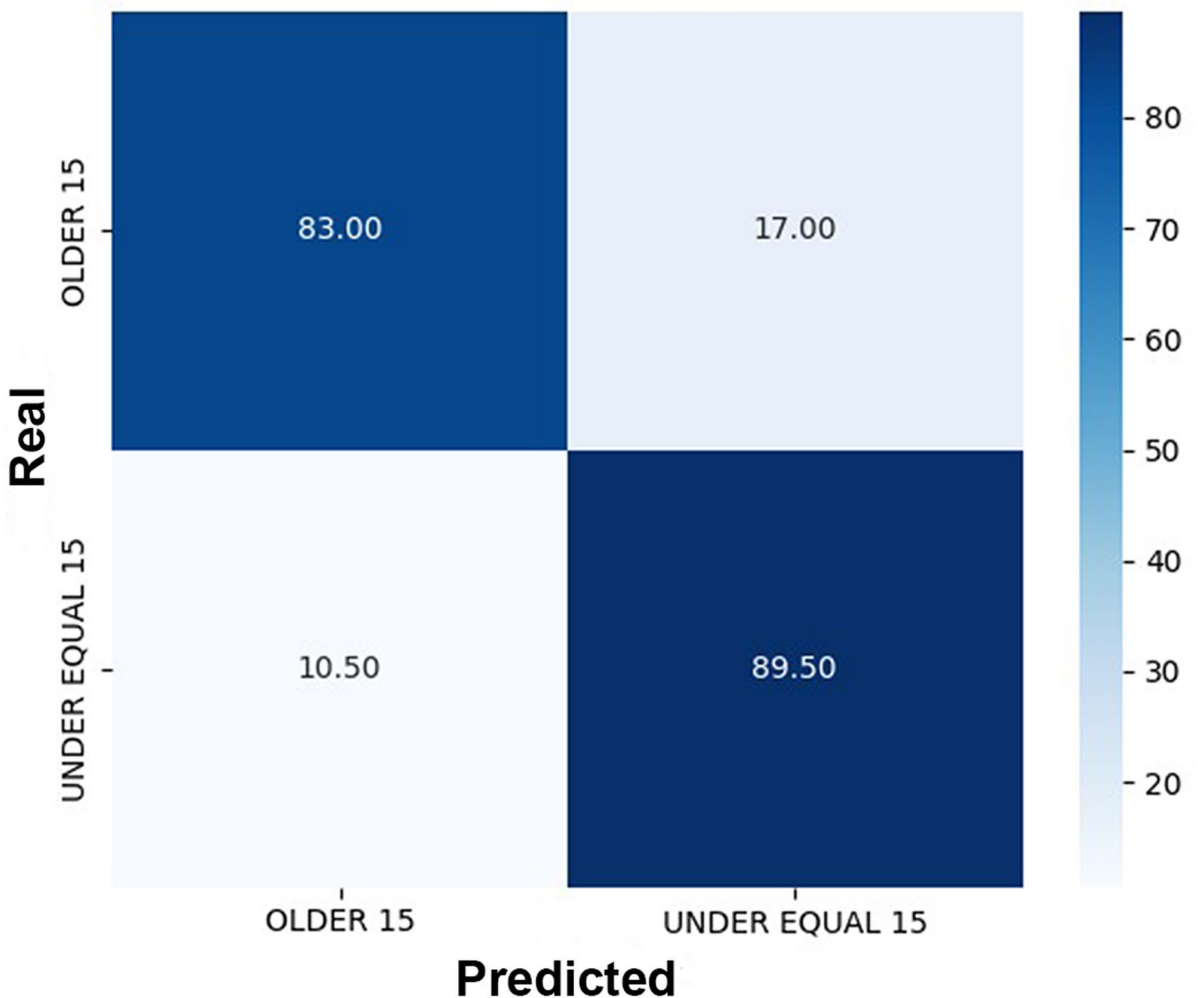
Confusion matrix comparing real (chronological) and predicted age of individuals divided into groups > 15 years and ≤ 15 years. The correct classification rate of individuals > 15 years was 83%, while for individuals ≤ 15 years it was 89.5%.

**Fig. 5:**
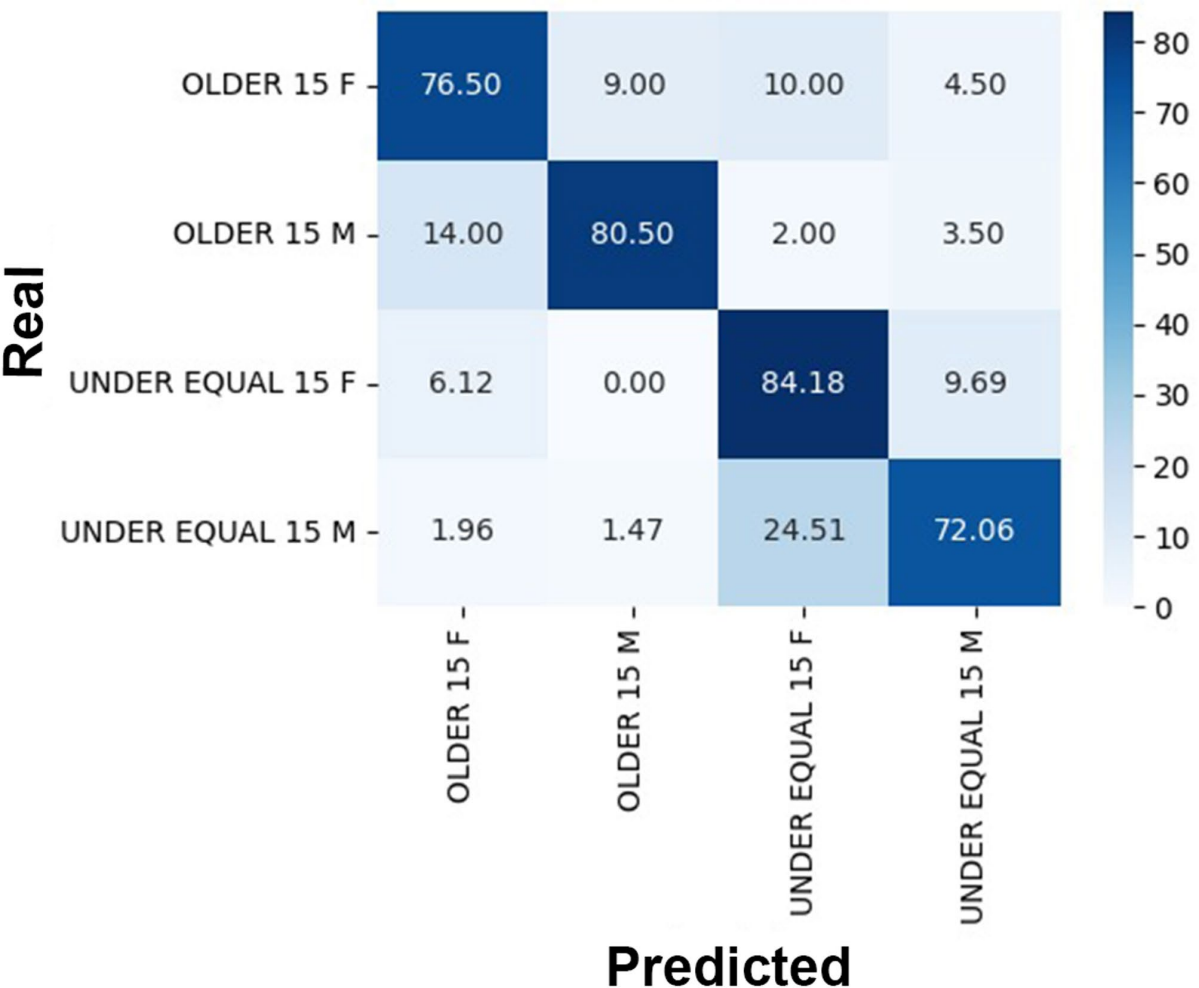
Confusion matrix comparing real (chronological) and predicted age of individuals divided into groups > 15 years and ≤ 15 years separately for males (M) and females (F). The correct classification rates of females and males > 15 years were 76.5% and 80.5%, respectively, while for females and males ≤ 15 years the results were 84.18% and 72.6%, respectively.

When evaluating the CNN’s performance in classifying individuals as below or above 15 years, the correct classification rate was 83% for those older than 15 years and 89.5% for those aged 15 years or younger (Fig. 4). The correct classification rate for males older than 15 years was 80.5% compared to 76.5% for females. Among individuals aged 15 years or younger, the correct classification rate was 72.06% for males and 84.18% for females (Fig. 5).

**Fig. 6:**
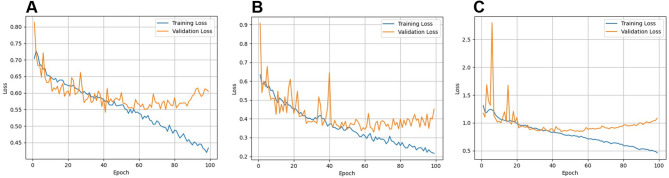
Loss function analysis over 100 epochs for sex (**A**), age (**B**) and the combined (**C**) classification tasks. Training (blue) and validation (orange) loss curves showing steadily decrease for the former and a plateaued-increase for the latter after 50 epochs.

Loss function analysis for sex, age and combined predictions demonstrated a pattern of relevant learning up to 50 epochs, with a plateau and subsequent divergence between training and validation curves (Fig. 6).

**Fig. 7:**
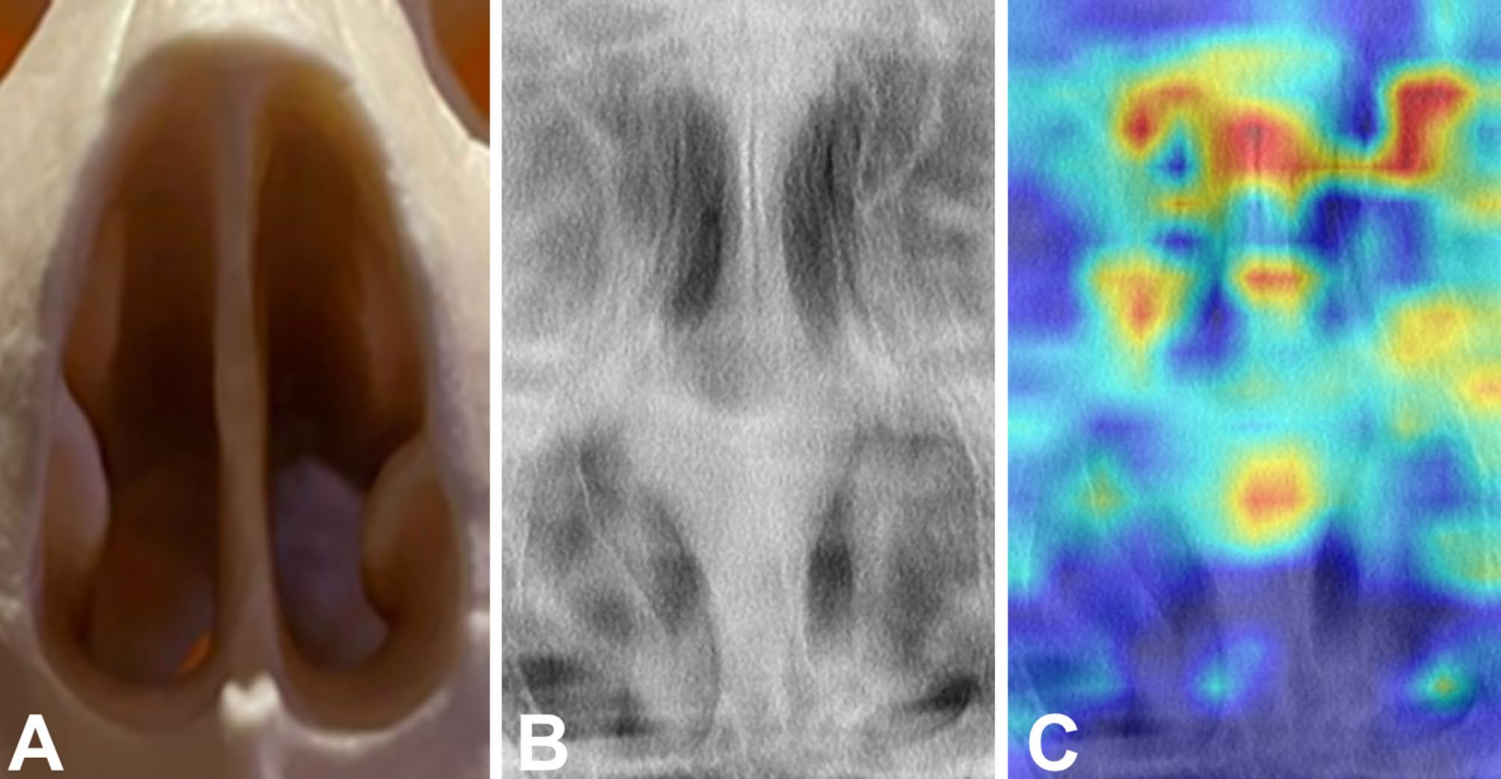
Nasal aperture (**A**), its region of annotation on panoramic radiograph (**B**) and the Gradient-weighted Class Activation Mapping (Grad-CAM) showing in red (**C**) regions of higher activation signal of the convolutional neural network to identify potential sex-related patterns during the classification task.

Grad-CAM analysis revealed stronger signals originating from the central mineralized tissue of the nasal aperture, including the nasal septum, as well as from the upper portion of the nasal aperture (Fig. 7).

## Discussion

Among the various morphological features of the viscerocranium, the nasal aperture is of special interest due to its potential applications in anthropological assessments of population affinity, sex, and age^32^. To date, studies on this topic have been conducted using morphometric^19,20,33,35^ and morphoscopic analyses,^21^ through direct examination of dry skulls^20,21,33^ or via medical imaging^19,35^. The latter has been conducted using imaging modalities that are either 3D,^19,34^ such as computed tomography scans, or 2D,^36^ such as extraoral radiography.

The present study revisited the topic using artificial intelligence solutions—specifically, CNN-based computer vision. A distinctive feature of the current methodological design was the sample composition, which included subadults (children and adolescents) as well as young adults. It is important to highlight that sex assessment in individuals under 12 years of age is generally not recommended due to a lack of reliable methods^36^. The rationale for the sample is based on the understanding that, while a sample composed entirely of mature specimens would yield more accurate sex estimates, including immature individuals in this diagnostic accuracy experiment allows for investigation of how age may influence the expression of morphological features between males and females. By doing so, the reduced expression of sexually dimorphic features at a young age was confirmed. For example, when the CNN’s sex estimation performance is analyzed separately for individuals under 12 years, the mean accuracy rate becomes 67%, representing a 10% decrease compared to individuals aged 12 years and older (Table 3). This decrease was even more pronounced among males, with a 14% drop. Interestingly, the accuracy rate observed using the 12-year threshold remained consistent when the threshold was raised to 15 years. In other words, accuracy rates remain considerably low even when the bony framework of the nose is expected to be more stable—after the age of 15 years, at least—^30^ compared to earlier immature phases. This finding underscores the importance of considering age^37^ when planning sex assessment and supports a previous study^21^ that suggested a possible absence of sexual dimorphism in the nasal aperture.

Authors have demonstrated morphological variance of the nasal aperture between populations^21^. In the present study, radiographs were sampled from an existing image database of the Central-West region, which likely included Brazilians with diverse populational affinities. A previous radiographic study with a Brazilian sample analyzed 97 individuals and found highly significant differences between males and females, with males exhibiting greater height, width, and area of the nasal aperture^35^. Our findings may differ based on methodology, including the larger sample size in the present study, the use of different extraoral imaging modalities, and the application of deep learning and image pattern analysis rather than the morphometric assessment of height, width, and area used by the previous authors^35^.

Compared to other maxillofacial features used for sex assessment, the nasal aperture has demonstrated inferior diagnostic accuracy. A study, applying landmarking to computed tomography scans, demonstrated up to 95% of sex classification accuracy after the analysis of adult human mandibles through machine learning. High accuracy rates have also been observed for statistical models based on the combination of cranial measurements – including the nasal aperture^38,39^. This phenomenon can be justified firstly by sample characteristics, covering only the adult age range, where sexual dimorphism can be more pronounced. Secondly, by the comprehensive approach of the human skull integrating several anatomic features that may express sexual differences. The mandible is an example of a bone that undergoes modeling and remodeling influenced by the surrounding musculature. Strong muscles, such as the masseters, insert onto the mandible and generate traction vectors in different directions. These biomechanical forces contribute to morphological changes and introduce variables that can be closely associated with sexual dimorphism. Other examples extend also to the posterior region of the skull, such as the sternocleidomastoid muscle and its influence on modeling the mastoid process region, possibly leading to differences^37^ between males and females.

To our knowledge, among the available studies assessing the nasal aperture, the present work is the first to apply CNN within an AI-based computer vision framework and includes the largest sample to date. As a result, a robust deep learning model was trained, contributing valuable insights to the scientific literature. Given the unsatisfactory accuracy rates and the high risk of misclassification, this study does not recommend using the nasal aperture as a reliable sexually dimorphic feature, especially considering population-specific variations. Moreover, careful interpretation is warranted when considering the study’s methodological approach to testing the CNN’s classification of age, which divided the sample into groups below or above the 15-year threshold. At first glance, the observed correct classification rates may seem promising; however, this setup posed a relatively simple task for the CNN—distinguishing radiographs of children as young as 6 years from young adults up to 22.9 years old using 15 years as the cutoff. Therefore, moderate to high correct classification rates were expected but offer limited practical applicability. Given the suboptimal correct classification rates and the frequency of misclassifications, alternative methods should be preferred to the nasal aperture for age assessment. In this regard, assessing permanent tooth development using CNN has shown to be a useful approach^7,10^.

In addition to the presented limitations, the type of imaging modality used in this study should also be considered. Panoramic radiographs, while common in extraoral imaging and often available in large datasets, capture the nasal aperture with the upper region typically positioned near the superior edge of the image. This positioning can cause the outline to appear smoothed or less defined. Moreover, the rotational acquisition technique employed in panoramic radiography provides a broad view of the maxillofacial structures but can also introduce^40^. To mitigate these limitations, the images were collected from an oral radiology center that follows standardized protocols. Additionally, pure morphometric analyses based on linear measurements—which can be biased in panoramic radiographs—were avoided by employing a computer vision approach that assesses image patterns. Future radiological studies should consider imaging modalities with fewer distortions, such as posteroanterior Caldwell radiography^35,41^ or Cone Beam Computed Tomography (CBCT). The latter allows for realistic assessment of the viscerocranium and dentomaxillofacial structures, along with 3D navigation^39^.

Another input for future studies is increasing the sample size and exploring not only a single CNN, but also alternative models. This is especially relevant because the training curves for all tasks showed a progressive reduction in training loss across epochs, indicating that the models were learning patterns from the radiographic data. However, the validation loss exhibited a distinct plateau followed by mild to moderate divergence from the training curve. This reflects a certain degree of overfitting, meaning that while the models continued to improve on the training data, their performance on unseen validation data stabilized or even worsened slightly around fifty epochs. Such behavior can be expected in deep learning applications with relatively limited sample sizes, where model capacity can exceed the available variability in the dataset. This finding corroborates the need for larger and alternative (focusing on sex estimation of adults, for instance) datasets to support stronger generalization, and testing additional architectures, regularization strategies, or hyperparameter adjustments in future research. Despite this, the models were still able to capture meaningful trends and provide results that support the feasibility of computer-aided approaches in forensic odontology, while making clear that expert confirmation remains indispensable.

In addition to these methodological perspectives, future research should also incorporate external validation to confirm the generalizability of our findings. External validation using independent datasets from different clinical centers is therefore important to test whether the patterns detected in the present study persist across populations, imaging protocols, and equipment. Such validation would not only demonstrate reproducibility and address potential biases linked to local features, but also clarify the extent to which computer-aided analysis of the nasal aperture can contribute to forensic applications, reinforcing that this structure alone is not a robust tool for definitive sex or age assessment.

## Conclusion

Under the present methodological conditions, the nasal aperture demonstrated limited discriminative power for sex classification, with performance metrics indicating an accuracy rate equivalent to one misclassification per four cases. A secondary, confirmatory finding was the notable drop in accuracy among younger individuals compared to older (mature) ones.

Therefore, the diagnostic accuracy metrics of the nasal aperture assessed from extraoral radiographs can be considered unsatisfactory to support its use as the sole anatomical feature for radiographic sex assessment via CNN.

## Supplementary Information

Below is the link to the electronic supplementary material.

Supplementary Material 1

## Data Availability

The data supporting this study’s findings are available from the project supervisor, Prof. Ademir Franco, upon reasonable request and with permission from the Center of Oral Radiology and Imaging.
